# Editorial Salutatory

**Published:** 1853-08

**Authors:** 


### EDITORIAL AND MISCELLANY.

An established usage requires that those who enter the editorial *sanctum* should perform certain acts of obeisance, and also after these manifestations of respect and readiness to serve, they should "declare their intentions" and make public their designs and motives. The first is altogether appropriate with those who are ready with these professions of "humble service," but we desire to be excused, because unskilled in the practice of these compliant flexures. We prefer plainness without ostentation, for,

"Treachery oft lurks
In compliment,"

and therefore we will proceed frankly to define ourselves in this our first *debut*, with the view to comply with the second requirement.

We have engaged in this work from an abiding sense of its necessity, and while we ask the patronage of a generous and an enlightened profession, we do so more for the sake of that profession—of Medical Science, than for ourselves. We enter upon the work profoundly assured that if *we* fail to interest and instruct, our enterprise must fail also, because the inherent merit of the work must constitute the element of its own success, nor could we consistently ask or expect patronage or support unless it prove worthy of it. This obtains where an undertaking of the kind is demanded by the wants and necessities of community, but if no such merit exist, then the most meritorious enterprise may fail.

We well know there is a need of such a Journal as the one we propose to publish and furnish to a profession, numerous enough in the State, united to neighboring districts in adjoining States, amply to sustain it. When this is done, it is all we crave, save a continuance of support. Our labor is theirs, freely, cheerfully—but gratuitously bestowed, for we assure our readers, we indulge in no hope of renumerative reward.

To the profession we would say that the "*Iowa Medical Journal*" is a thing that *is now* and *will be*. We shall continue its publication at all hazards, and at whatever reasonable sacrifice, and the medical public is assured that just *so long* we will relax no effort, but exert all our power to make it useful, interesting, and profitable. We enter the lists with those now engaged in the advocacy and defence of the truths of the science and of true legitimate medicine. We shall, to the utmost of our feeble abilities, maintain its dignity and promote its acknowledged usefulness and efficiency. And, while in this path of duty we crave all the light which can be rendered us by our medical brethren, yet we will not be seduced from our present design, to present facts both useful and instructive, by deceptive prismatic lights, however glittering and bright, liable to change color with every change of the prism.

We are aware to some extent, of the labors and difficulties which lie before us, but at the same time we are as fully assured that when engaged in a useful and praise-worthy undertaking, energy, persevering effort, and an untiring devotion, will ultimately triumph; and with the kind encouragement of the profession, after justly appreciating our motives, *triumph we will.* We have the will, with *them* resides the power. If the last be exercised, we pledge ourselves to make the Journal behind none other in the West in usefulness and excellence, proportionate to its size. We then ask our friends in the profession, and others desirous of aiding this enterprize in Medical literature, to send on their names. We have already a number upon our list, not only as subscribers but as contributors, of whom we are proud, as they rank among the oldest of the profession in this country. We have subscribers now from the States of Illinois, Missouri, Ohio and this State. We are cheered by the encouragement already received even before a number is published, and hope that the list of subscribers will be speedily augmented.

The title of the work we hope will meet with favor. We desire to make it what its name implies, the organ of the profession of the State. Bearing its name we desire to infuse into its pages vigor and energy, elements and agents now active in developing the State's vast and exhaustless resources and soon to elevate her to a position and rank with other States, as among the first and most enlightened of the Union. She has fully escaped from her infancy with all its attendant dependencies, and is now a cherry-cheeked maiden, vigorous and blooming in her teens, with smiles of hope and happy assurances upon her bright face. All her departments of usefulness and utility are advancing to comparative perfection, whether agricultural or mechanical, literary or scientific, among which last medical science stands prominently forward, but like every other department, it must be fostered and sustained. For this purpose and for this object this work is established and will be continued. To our medical brethren we freely entrust our undertaking with a confidence which finds its basis in the hallowed feeling and sympathy subsisting between honorable men, honestly engaged in kindred pursuits, and anxious to sustain the scientific institutions of the country.

The prompt appearance of our Journal every month will be a great and important element in its usefulness and efficiency. Vigilance is as requisite in medical science, as its constant exercise is necessary to the perpetuation of liberty. Important discoveries and new facts cannot too soon be disseminated among medical men. In times when a pestilence is making its dread march over the land, every newly discovered fact in its pathology or treatment should, as if by one trumpet-blast, be spread all over the country, penetrating into every locality where the destroyer may have entered. The number of Medical Journals and other publications, foreign and American, appearing at different times and in rapid succession, will enable us to select weekly from various sources, and to cull the choicest fruits in medical literature for our monthly contributions, and thereby to minister to the wants and gratification of the medical mind of the State and our readers every where.

A newly developed truth in the science is not the sole property of the originator, but in obedience to the wants and the spirit of humanity, it is called forth and appropriated to the benefit of mankind, while the author's name is blended with and ranks in prominence, and importance with, the discovery itself. In proportion to the benefits it confers and to that degree, does his name become exalted among men. But the benefits of which it is susceptible, are not his to limit or extend at his will or pleasure, for the discovery would cease to possess the element of excellency as long as it was confined to his own bosom or allowed but a partial range of good. To constitute a great good it must be universal and the earlier it is disseminated, the sooner will it possess this attribute. The untold benefits conferred by vaccination has made the name of Jenner immortal, because they have a diffusion coequal with civilization, and it possesses the attribute of excellency because almost universal in its use. Yet while his name will live in the grateful remembrance of civilized men and his memory hallowed by his discovery, still humanity claimed the abstract benefits as hers and requested that the discovery itself should be *speedily* and widely disseminated.

